# Basal and therapy-driven hypoxia-inducible factor-1α confers resistance to endocrine therapy in estrogen receptor-positive breast cancer

**DOI:** 10.18632/oncotarget.3257

**Published:** 2015-04-13

**Authors:** Xiaoqing Jia, Qi Hong, Li Lei, Daqiang Li, Jianwei Li, Miao Mo, Yujie Wang, Zhimin Shao, Zhenzhou Shen, Jingyi Cheng, Guangyu Liu

**Affiliations:** ^1^ Department of Breast Surgery, Key Laboratory of Breast Cancer in Shanghai, Fudan University Shanghai Cancer Center, Department of Oncology, Shanghai Medical College, Fudan University, Shanghai, P.R. China; ^2^ Department of Breast Surgery, The First People's Hospital of Kunming, Kunming, P.R. China; ^3^ Clinical Statistics Center, Fudan University Shanghai Cancer Center, Department of Oncology, Shanghai Medical College, Fudan University, Shanghai, P.R. China; ^4^ Department of Nuclear Medicine, Fudan University Shanghai Cancer Center, Department of Oncology, Shanghai Medical College, Fudan University Shanghai, P.R. China

**Keywords:** HIF-1a, endocrine resistance, zoledronic acid, estrogen receptor, breast cancer

## Abstract

Resistance is an obstacle to endocrine therapy for breast cancer. We measured levels of hypoxia-inducible factor (HIF)-1α in 52 primary breast cancer patients before and after receiving neoadjuvant endocrine therapy with letrozole for at least 3 months. Pre-treatment levels of HIF-1α were associated with negative clinical outcome. Furthermore, levels of HIF-1α were increased in post-treatment residual tumors compared with those in pre-treatment biopsy samples. In animal studies, xenografts stably expressing HIF-1α were resistant to endocrine therapy with fulvestrant compared with the effects in control xenografts. Additionally, HIF-1α transcription was inhibited by zoledronic acid, a conventional drug for the treatment of postmenopausal osteoporosis, and was accompanied by a marked inhibition of the RAS/MAPK/ERK1/2 pathway. HIF-1α is a determinant of resistance to endocrine therapy and should be considered as a potential therapeutic target for overcoming endocrine resistance in estrogen receptor (ER)-positive breast cancer. In addition, zoledronic acid may overcome endocrine resistance in ER-positive human breast cancer by targeting HIF-1α transcription through inhibition of the RAS/MAPK/ERK1/2 pathway. Clinical studies on the administration of zoledronic acid as a second line treatment in patients who failed endocrine therapy should be considered to improve therapeutic outcomes in breast cancer patients.

## INTRODUCTION

Approximately 70% of breast carcinomas are hormone-dependent and estrogen receptor (ER) positive [1]. Patients with this type of breast cancer are candidates for endocrine therapy, however, a number of patients will develop acquired resistance to endocrine therapy after initial treatment, and nearly 50% of advanced ERα-positive breast cancer patients do not respond to tamoxifen or aromatase inhibitors (AIs) in the first-line treatment. However, the mechanism underlying acquired therapeutic resistance remains elusive.

The cellular response to hypoxia involves the increased expression and activity of HIF-1α, which regulates a large subset of target genes essential for cellular adaptation to low oxygen conditions [2]. Under normoxic conditions, HIF-1α is modified at the proline residues (pro564 and pro402) by prolyl hydroxylases and targeted for ubiquitination and degradation by interacting with the von Hippel–Lindau tumor suppressor protein (VHL), which is a specific substrate-recognition component of the E3 ubiquitin complex [3]. Under hypoxic conditions, the HIF-1α protein is stabilized through the inactivation of an oxygen-dependent HIF-1α-prolyl hydroxylase, and then, translocates to the nucleus, where it dimerizes with the HIF-1β subunit [4, 5]. Several studies have indicated that HIF-1α expression is strongly associated with tumor initiation, malignant progression, and resistance to radiotherapy and chemotherapy [6–8].

Recently, it was found that hypoxia significantly reduced the growth-promoting effect of estradiol (E2) and the growth-inhibitory effect of an anti-estrogen drug [9]. Furthermore, a recent clinical study comparing the effect of neoadjuvant letrozole with that of letrozole plus metronomic cyclophosphamide on tumor growth inhibition revealed that increased HIF-1α expression significantly predictive of therapeutic resistance [10]. However, there is still a lack of direct *in vivo* evidence to establish the relationship between HIF-1α expression and endocrine resistance. Moreover, whether HIF-1α acts as a driver in the development of endocrine resistance, or simply as one of the markers indicating hypoxia within the tumor remains to be clarified. The aim of this current study was to examine the association of endocrine resistance in human breast cancer with hypoxia and its major regulator, HIF-1α, *in vivo*.

Zoledronic acid is the standard therapy for patients with bone metastasis and osteoporosis [11]. Recent clinical studies have shown that adding zoledronic acid to endocrine therapy significantly improves patient-survival [12, 13]. The direct anti-tumor effect of zoledronic acid has also been shown in other preclinical studies, in which zoledronic acid inhibited proliferation, invasion and metastasis of tumors in addition to promoting tumor cell apoptosis [14–16]. Furthermore, the results of the ABCSG-12 and ZO-FAST trials clearly support the potential anticancer activity of zoledronic acid [12, 13].

In this study, we investigated the hypothesis that HIF-1α expression contributes to the resistance to endocrine therapy in breast cancer. We generated MCF-7 breast cancer cells stably expressing HIF-1α (MCF-7/HIF-1α), which were resistant to endocrine therapy and found that targeting HIF-1α reversed endocrine resistance both *in vitro* and *in vivo*. These data provide evidence of the involvement of HIF-1α in breast cancer endocrine resistance. Targeting HIF-1α by zoledronic acid effectively reversed endocrine resistance, which supports the preclinical and clinical development of a novel therapeutic strategy to overcome HIF-1α driven resistance to anti-estrogen therapy.

## RESULTS

### Baseline HIF-1α expression was negatively correlated with clinical outcome in the neoadjuvant endocrine therapy group

In total, we recruited 52 postmenopausal patients with stage II–III ER-positive primary breast cancer who consented to receive primary endocrine therapy with letrozole (Femara 2.5 mg daily). Sixteen (30.8%) patients obtained a clinical response (complete response [CR] + partial response [PR]) in their primary lesions after at least 3 months of treatment.

**Figure 1:**
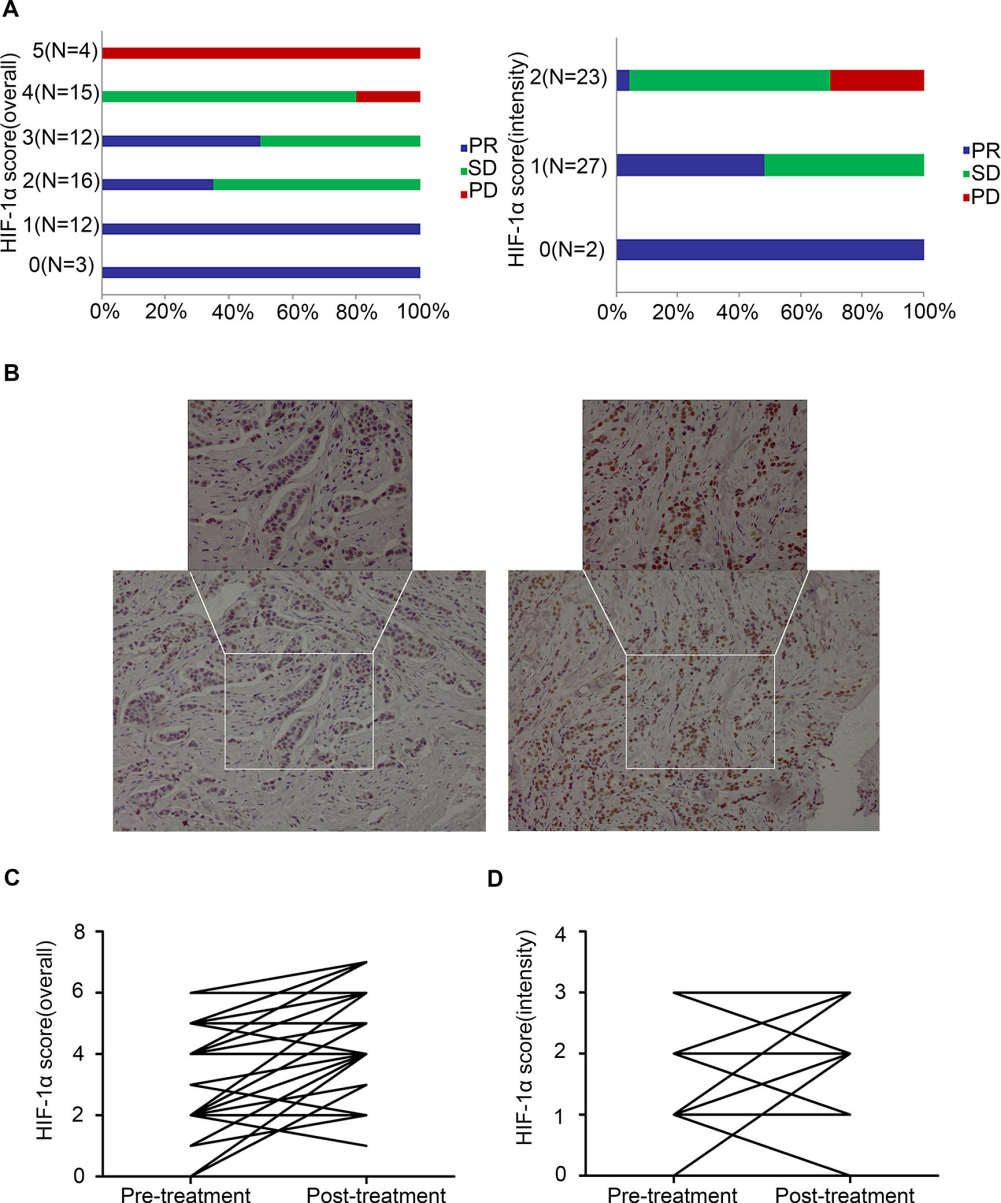
Correlation between pre-treatment (baseline) HIF-1α expression and the clinical objective response to primary endocrine therapy (*n* = 52, *p* < 0.0001, Chi-square test) **(A)** Correlation between pre-treatment HIF-1α expression (overall score and intensity score) and the clinical objective response to primary endocrine therapy; **(B)** IHC detection of HIF-1α expression of case No. 35 at baseline and post-treatment (20 ×, 40 ×); **(C)** Comparison of HIF-1α overall score (combined proportion and intensity) between baseline and post-treatment samples in the whole set; **(D)** Comparison of HIF-1α intensity score between baseline and post-treatment samples in the whole set.

In the subset of 52 patients, baseline HIF-1α expression was detected by IHC analysis (Supplementary Figure 2). A good correlation was observed between pre-treatment HIF-1α expression (overall score and intensity score) and clinical outcomes (*p* < 0.001, Chi-square test, Figure 1A).

### Increased HIF-1α expression is associated with resistance to primary endocrine therapy in primary breast cancers

In the subset of 52 patients, HIF-1α expression levels before and after treatment were compared based on IHC analysis (Figure 1B). The HIF-1α expression level in the post-treatment samples was significantly increased compared with that at baseline, regardless of the scoring methods applied (overall score or intensity score, *p* < 0.0001 or *p* = 0.0002, respectively; Paired *t*-test), and such changes were also consistent regardless of the response types: partial response (PRs), stable disease (SDs) or progressive disease (PDs) (Figure 1C, 1D).

### HIF-1α is involved in endocrine resistance in ER-positive MCF-7 cells

**Figure 2:**
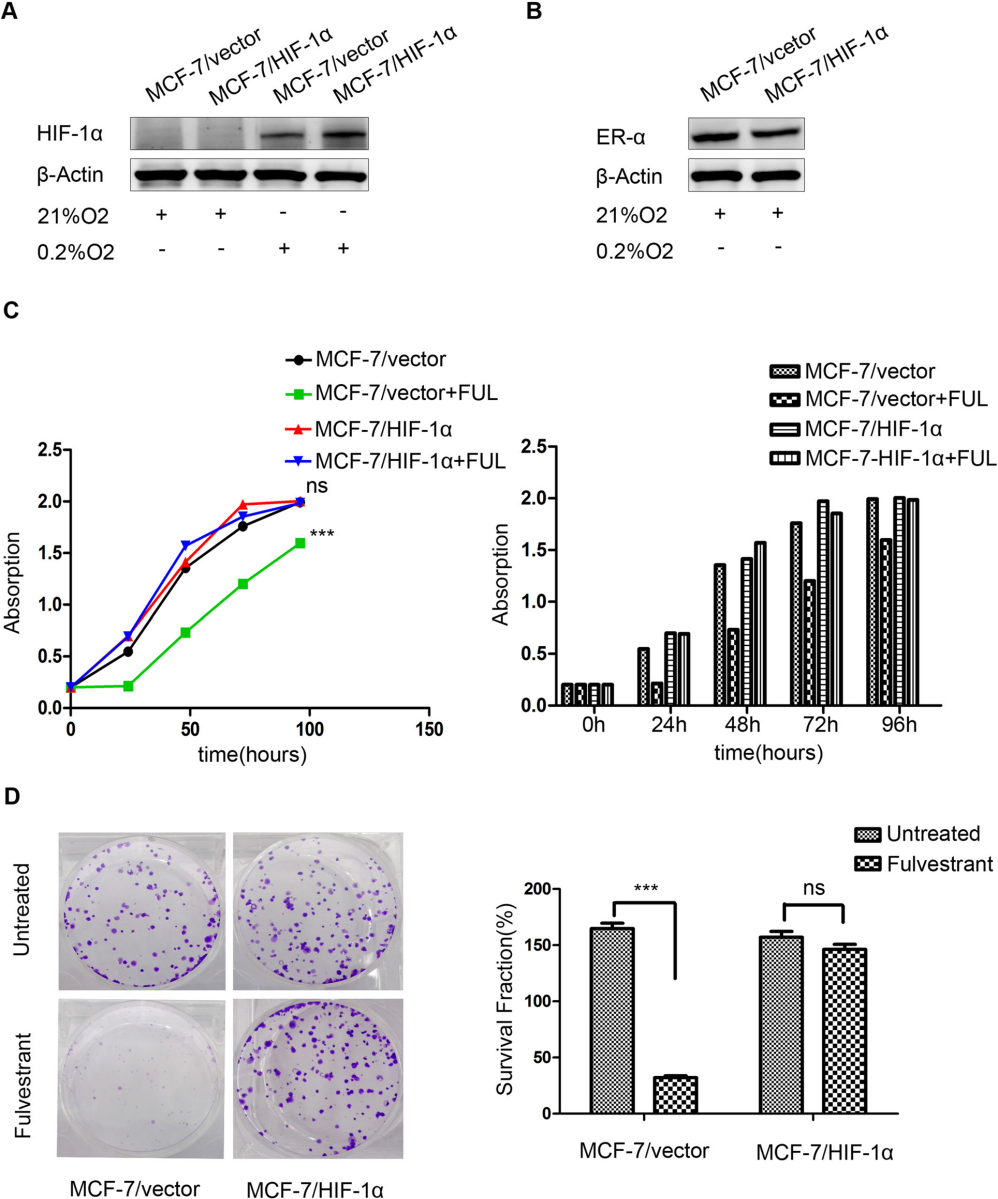
Stable over-expression of HIF-1α decreases the sensitivity of breast cancer cells to fulvestrant **(A)** HIF-1α expression was higher in MCF-7/HIF-1α cells than that in MCF-7/vector cells under hypoxic conditions; **(B)** ERα expression was not significantly changed in MCF-7/HIF-1α cells compared to that in MCF-7/vector cells under normoxic conditions; **(C)** Fulvstrant-treated MCF-7/HIF-1α cells showed no detectable difference after drug treatment, whereas MCF-7/vector cells exhibited substantially slower growth compared to untreated cells; **(D)** MCF-7/HIF-1α cells formed significantly more colonies than did MCF-7/vector cells after fulvestrant treatment.

To explore the potential role of HIF-1α in endocrine resistance, we established MCF-7 cells stably expressing vector control (MCF-7/vector) or HIF-1α (MCF-7/HIF-1α). HIF-1α proteins are rapidly degraded and undetectable under normoxic conditions, therefore, we detected HIF-1α expression under hypoxic conditions. As expected, Western blot analysis revealed that HIF-1α expression levels were significantly increased in MCF-7/HIF-1α cells compared with those in MCF-7/vector cells under hypoxic conditions, but not under normoxic conditions (Figure 2A). MCF-7/HIF-1α remained ERα-positive status under normoxic conditions (Figure 2B). Fulvstrant significantly inhibited the growth of MCF-7/vector cells but not MCF-7/HIF-1α cells (Figure 2C). Moreover, the colony formation assay also showed consistent results (Figure 2D).

### Effect of HIF-1α on xenograft tumor formation and sensitivity to anti-estrogen treatment in mice xenografts

**Figure 3:**
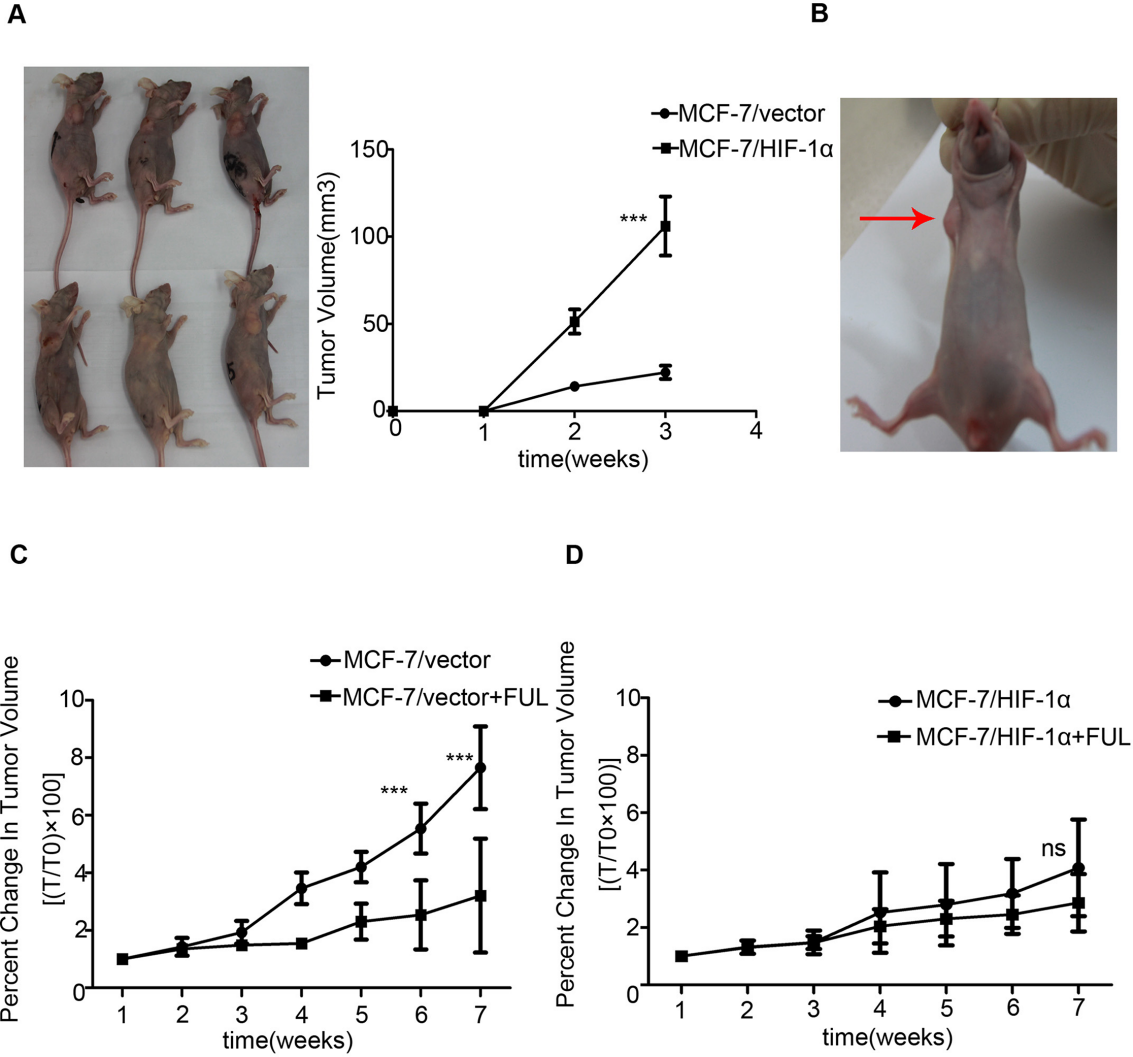
Stable over-expression of HIF-1α decreases the sensitivity of xenograft to fulvestrant **(A)** MCF-7/HIF-1α cells exhibited quicker and larger xenograft formation compared to the corresponding control groups; **(B)** MCF-7/HIF-1α cells exhibited larger xenograft formation compared to those formed by MCF-7/vector cells in one nude mouse; **(C)** The drug-sensitive MCF-7/vector xenograft tumor volumes were significantly reduced. **(D)** The growth of drug-resistant MCF-7/HIF-1α tumors was not affected by fulvestrant treatment.

To further investigate the effect of HIF-1α on anti-estrogen treatment *in vivo*, MCF-7/HIF-1α and MCF-7/vector cells were used to establish a xenograft tumor model. MCF-7/HIF-1α cells exhibited quicker and larger xenograft tumor formation than those of formed by MCF-7/vector cells (Figure 3A). To further clarify the differences in the formation of tumors between MCF-7/HIF-1α and MCF-7/vector cells, we established a mouse model in which MCF-7/HIF-1α and MCF-7/vector cells were inoculated simultaneously in the right and left fat pads of the same nude mouse. The results showed that MCF-7/HIF-1α cells exhibited larger xenograft tumor formation compared with those formed by MCF-7/vector cells (Figure 3B). Following fulvestrant treatment, the volumes of the drug-sensitive MCF-7/vector xenograft tumors were significantly smaller compared to those formed in the MCF-7/HIF-1α groups (Figure 3C). In contrast, the growth of drug-resistant MCF-7/HIF-1α tumors was not affected (Figure 3D).

### HIF-1α may be a crucial determinant of endocrine resistance in MCF-7 breast cancer cells

**Figure 4:**
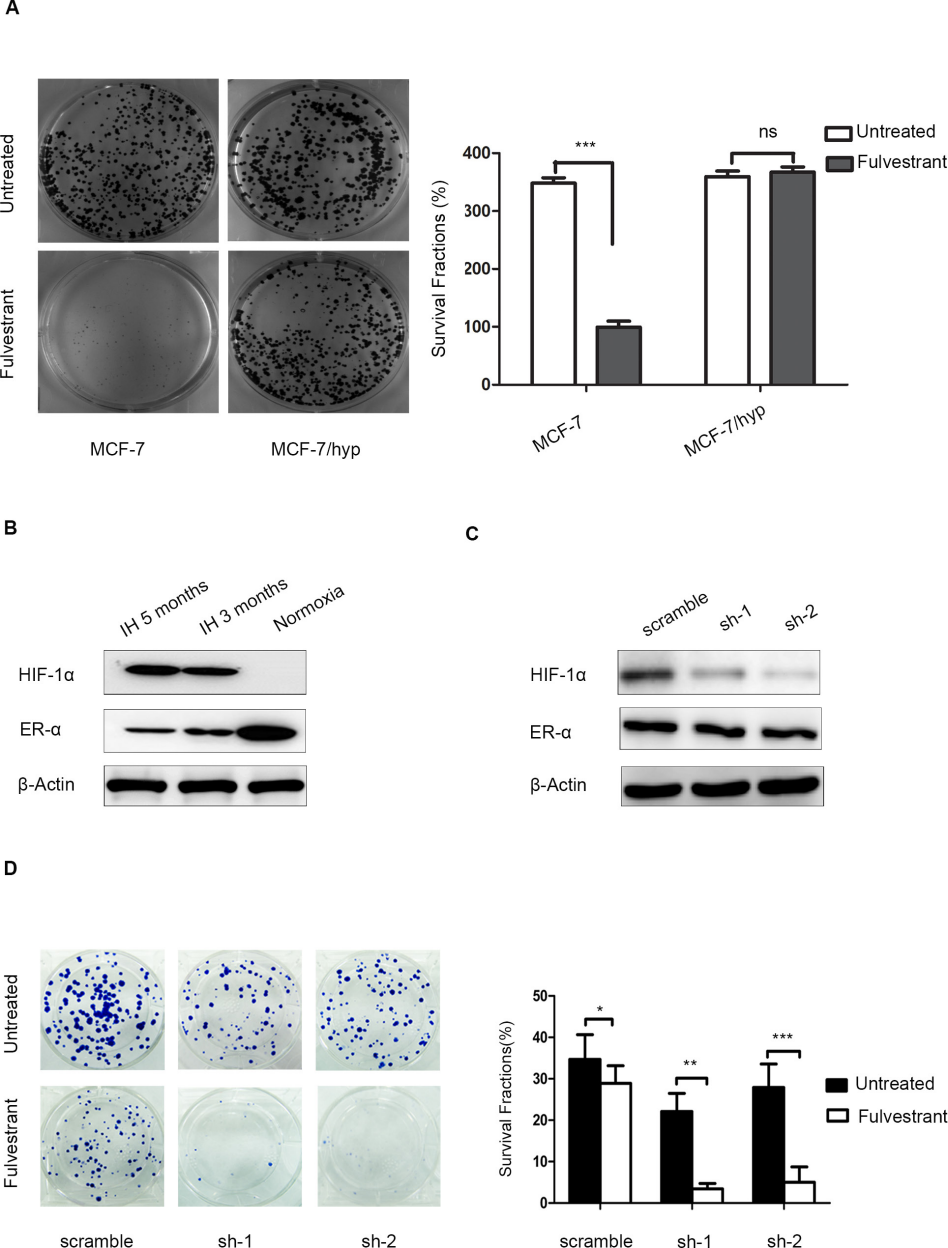
Knockdown of HIF-1α expression in intermittent hypoxic cells sensitizes breast cancer cells to fulvestrant **(A)** MCF-7/hyp cells formed significantly more colonies than did wild-type MCF-7 cells after anti-estrogen fulvestrant treatment; **(B)** Western blot analysis showed activation of HIF-1α expression and maintenance of ERα expression in MCF-7/hyp cells, even after their return to a normoxic environment; **(C)** MCF-7-hyp cell lines stably expressing two different shRNAs targeting HIF-1α (sh-1 and sh-2) and the control cell line with scramble shRNA (scramble) were established. Western blot analysis showed expression of HIF-1α was significantly down-regulated in sh-1 and sh-2 cell lines; **(D)** Cell proliferation was inhibited more significantly by fulvestrant in sh-1 and sh-2 cell lines (*p* < 0.0001 and *p* = 0.0001 respectively) compared with the scramble control.

To elucidate the intrinsic relationship between HIF-1α and the endocrine responsiveness of ER-positive breast cancer cells, we developed an MCF-7 cell line pre-cultured in a long-term and intermittent hypoxic environment (MCF-7/hyp). MCF-7/hyp cells were less sensitive to anti-estrogen treatment, compared with the wild-type MCF-7 cells (Figure 4A). To investigate the potential role of HIF-1α and ERα in HIF-1α-induced endocrine resistance, we assessed the levels of HIF-1α and ERα protein expression in MCF-7/hyp cells and MCF-7/wt cells *in vitro* by Western blot analysis. As shown in Figure 4B, detectable levels of HIF-1α expression and maintenance of ERα expression were observed in MCF-7/hyp cells, even if they were returned to a normoxic environment. The role of HIF-1α in endocrine resistance of breast cancer cells was further investigated by shRNA-mediated knock down of HIF-1α expression in MCF-7/hyp cells. Expression of HIF-1α was significantly down-regulated in stable HIF-1α knockdown MCF-7/hyp cells compared with the controls (Figure 4C). As shown in Figure 4D, cell proliferation was more significantly inhibited by fulvestrant in HIF-1α knock-down MCF-7/hyp cells (*p* < 0.0001 and *p* = 0.0001, respectively) as compared with the scramble controls.

### Zoledronic acid mediates partial inhibition of HIF-1α expression through the RAS/MAPK/ERK1/2 signaling pathway

**Figure 5:**
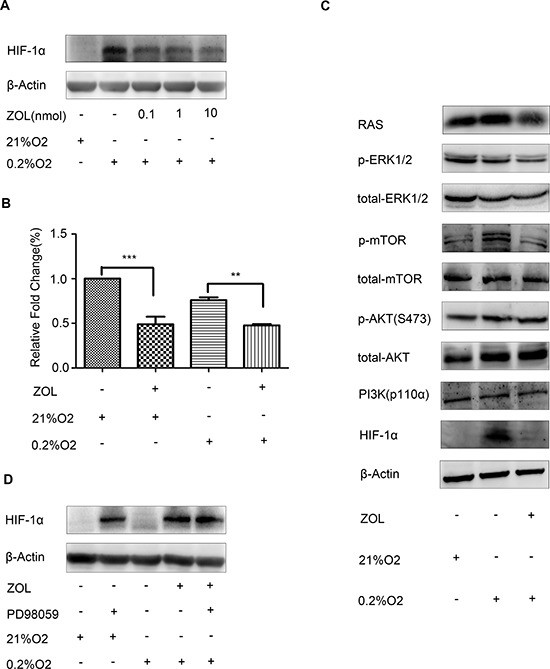
Effect of zoledronic acid on HIF-1α expression in MCF-7 cells **(A)** MCF-7 cells were cultured under 21% or 0.2% O_2_ for 2 h prior to treatment with various concentrations of zoledronic acid (0.1, 1, 10 μM) for an additional 16 h. Control cells received an equal volume of dimethyl su lfoxide. Immunoreactive bands were quantified against β-actin and presented as relative optical density. Data from three experiments are summarized in the lower panel; the *p*-values were calculated according to Student's *t*-test. **(B)** Real-time-PCR analysis of the relative gene expression level of HIF-1α in MCF-7 cells grown under 0.2% or 21% O_2_ for 2 h prior to the addition of zoledronic acid (10 μM) for further 16 h. Data represent the mean of triplicate samples and *p*-values were calculated using Student's *t*-test. **(C)** Western blot showing that zoledronic acid significantly inhibited phosphorylated-ERK1/2, but had no obvious effects on phosphorylated-AKT and PI3K (p110α) levels. **(D)** Zoledronic acid showed no obvious effects on HIF-1α protein accumulation following pretreatment with PD98059, a specific inhibitor of ERK1/2.

It has been reported that bisphosphonate acids, such as zoledronic acid, inhibit expression of HIF-1α in breast cancer cells [17]. To explore this effect, MCF-7 cells were subjected to hypoxia and treated with zoledronic acid for 16 h. Our results showed that zoledronic acid inhibited HIF-1α protein expression (Figure 5A). To further investigate whether the inhibition of HIF-1α expression by zoledronic acid was the result of transcriptional inhibition, we evaluated the levels of HIF-1α mRNA by real-time PCR. As shown in Figure 5B, treatment with zoledronic acid significantly inhibited HIF-1α mRNA expression both under normoxic and hypoxic conditions. Taken together, these results suggest that zoledronic acid inhibit the expression of HIF-1α at the level of transcription.

Previous studies have shown that HIF-1α is activated in a RAS-dependent manner [18], and that the PI3K/Akt and ERK1/2 signaling pathways are involved in HIF-1α protein expression [19]. To elucidate the mechanism by which zoledronic acid inhibits HIF-1α expression, we next examined the effect of zoledronic acid on the activity of RAS-dependent signaling pathways. Interestingly, we found that zoledronic acid significantly reduced phosphorylated-ERK1/2 levels, but had no obvious effects on phosphorylated-AKT and PI3K (p110α) levels (Figure 5C). When pre-treat with PD98059, a specific inhibitor of ERK1/2, zoledronic acid showed no obvious effects on HIF-1α protein expression (Figure 5D). Our results indicate that zoledronic acid inhibited HIF-1α expression, at least in part, through the RAS/MAPK/ERK1/2 signaling pathway.

### Zoledronic acid decreases HIF-1α expression in patients with ER-positive breast cancer receiving primary endocrine therapy

**Figure 6:**
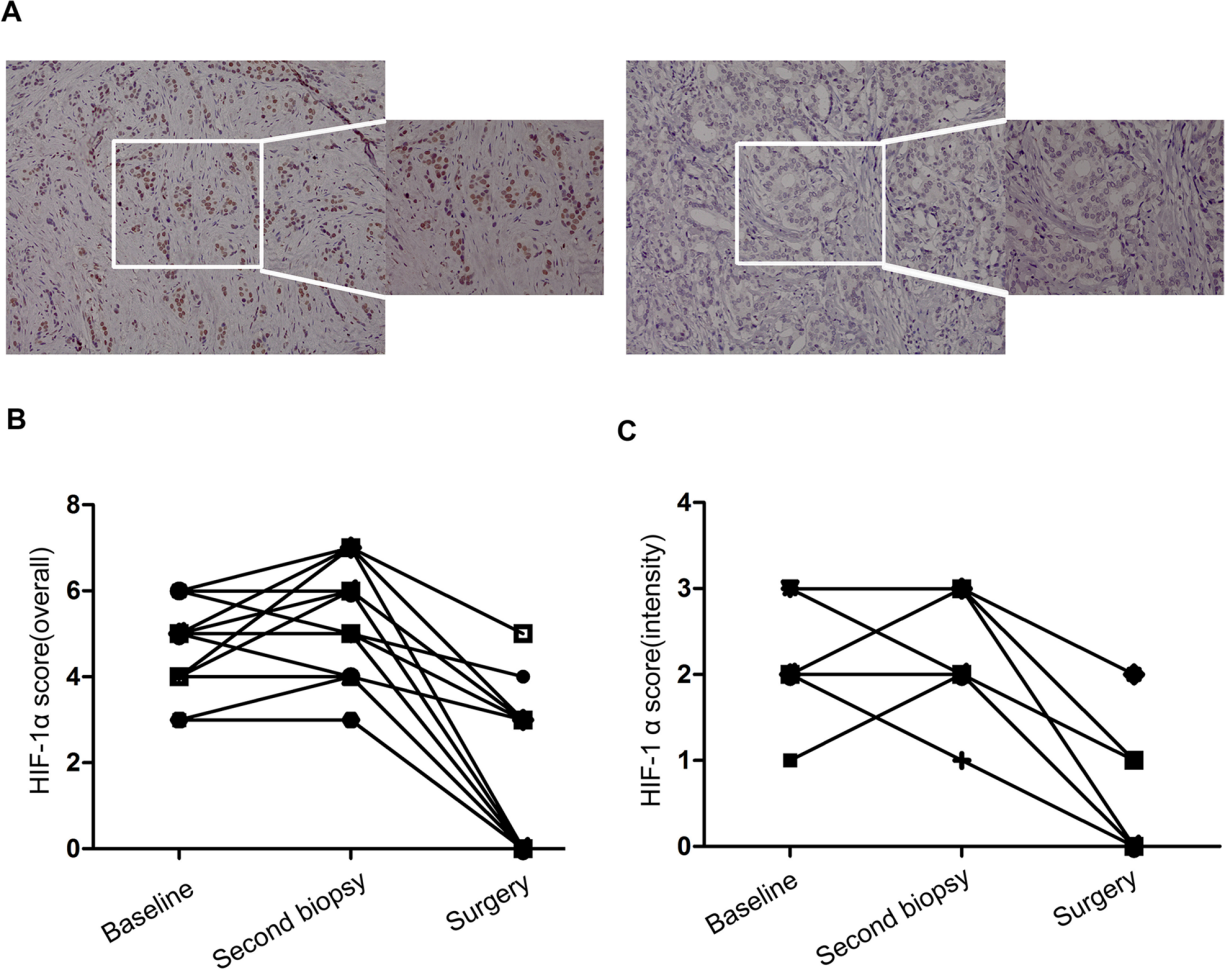
The effect of zoledronic acid combined with letrozole on endocrine therapy sensitivity *in vivo* **(A)** Immunohistochemical analysis of HIF-1α expression in pre- and post-zoledronic treatment samples (case No. 26, 20 ×, 40 ×); **(B)** Comparison of HIF-1α overall score (combined proportion and intensity) between pre- and post-zoledronic treatment samples in the whole set; **(C)** Comparison of HIF-1α intensity score in pre- and post-zoledronic treatment samples in the whole set.

In another subset of 20 patients, we carried out IHC analysis of the HIF-1α expression levels before and after adding zoledronic acid to primary endocrine therapy. HIF-1α expression levels were examined by IHC staining of tissue samples at three time points: before primary endocrine therapy, after at least 3 months of endocrine therapy, and 4 weeks after adding zoledronic acid to endocrine therapy (Table 1). HIF-1α levels were observed after initiating primary endocrine therapy for at least 3 months in this subgroup of patients (Figure 6A). In contrast, a statistically significant decrease (*p* < 0.0001) in HIF-1α expression (overall score and intensity score, *P* < 0.0001; Paired *t*-test) (Figure 6B, 6C) was observed just 4 weeks after adding zoledronic acid in this subgroup of patients.

**Table 1:** Baseline and second core needle biopsy (pre-zoledronic acid treatment) and surgery (post-zoledronic acid treatment) immunohistochemistry (IHC) scores of HIF-1α staining and clinical outcomes of primary endocrine therapy in primary breast carcinoma patients

Patients	HIF-1α (score)			Clinical response
	Baseline	Second biopsy	Surgery	
1	5	5	4	SD
2	3	4	3	SD
3	5	5	0	PD
4	4	6	3	PR
5	5	7	0	PD
6	6	6	0	SD
7	4	7	5	SD
8	4	4	0	SD
9	5	5	0	PR
10	5	7	3	SD
11	6	7	3	SD
12	6	5	0	SD
13	5	5	0	SD
14	6	5	0	PR
15	5	5	0	SD
16	5	6	0	PR
17	5	4	3	SD
18	5	5	3	SD
19	3	3	0	PR
20	5	6	0	SD

Abbreviations: HIF, hypoxia inducible factor; PR, partial response; SD, stable disease; PD, progressive disease.

## DISCUSSION

Despite advances in understanding of ER-positive breast cancer, endocrine resistance is yet to be overcome. Although numerous studies have been conducted to explore the mechanisms of endocrine resistance, which may be correlated with changes in ER structure and function and crosstalk with the epidermal growth factor receptor signaling pathway [20, 21], endocrine resistance remains a challenge. Understanding other mechanisms of anti-estrogen resistance may help in the identification of targets whose inhibition to restore drug responses and provide new treatment options for breast cancer.

As observed in this study, HIF-1α is overexpressed in the majority of advanced breast cancers [22]. Previous studies have shown that HIF-1α is strongly associated with tumor propagation, malignant progression, and resistance to radiotherapy and chemotherapy [6–8, 23]. Many studies have focused on new drugs targeting the HIF-1α pathway. For example, inhibition of HIF-1α by YC-1 decreased proliferation and metastasis in breast cancer [24]. In addition, anti-angiogenic therapy is an effective approach, although, the associated hypoxia may drive tumor progression and metastasis [25–27]. Blagosklonny reviewed the relationship between anti-angiogenic therapy and tumor progression [28]. Under hypoxic conditions, HIF-1α is stabilized, rapidly accumulates and transactivates various genes, including angiogenic genes. It also inhibits secretion of anti-angiogenic factors, such as thrombospondin-1 (TSP-1), thus stimulating angiogenesis [29–31], and resolving hypoxia. Consequently, it can be speculated that anti-angiogenic therapy may promote metastasis and invasion by activating the hypoxic response in cancer cells [25–27]. In the clinic, however, anti-angiogenic therapy has not been shown to induce metastases [32]. Therefore, only successful anti-angiogenic therapy, which is capable of controlling cancer, will select for resistance and progression.

Generali et al found that increased HIF-1α levels were significantly predictive factors of resistant to endocrine therapy [10, 33]. Our previous study showed that ^18^Fmiso-uptake in hypoxic malignant lesions could be used to predict primary endocrine therapy resistance [34], suggesting that hypoxia and HIF-1α play a critical role in endocrine resistance. However, the causal relationship between HIF-1α and endocrine resistance of human breast cancer *in vivo* remains controversial. One possibility is that increased HIF-1α expression facilitates endocrine resistance of breast cancer cells due to accelerated proliferation of the uncontrolled cancer cells and a lack of blood supply. However, this is not supported by our observation in human breast cancer cells *in vitro* and *in vivo* studies on nude mice, which showed that the expression of HIF-1α in the residual tumors is enhanced not only in poor-responders to primary endocrine therapy, but also in good-responders. It can be speculated that a more reasonable explanation for this phenomenon is that, under hypoxic conditions, a subgroup of cancer cells expressing high levels of HIF-1α lose their hormone sensitivity and selectively survive after primary endocrine therapy. To confirm this hypothesis, we developed the HIF-1α stably expressing ERα-positive human breast cancer cell line, MCF-7/HIF-1α, and successfully established xenografts in nude mice. Compared with the control cell line, MCF-7/hyp cells display an increased HIF-1α expression, a higher potential for tumor formation and less sensitivity to the anti-estrogen agent, fulvestrant. Our results indicated that HIF-1α plays a vital role in endocrine resistance, and should be considered as a future therapeutic target for overcoming endocrine resistance in ER- positive breast cancer.

Zoledronic acid is a nitrogen-containing bisphosphonate, which attaches to the mineralized bone matrix and is ingested by osteoclasts during osteolysis, thereby inhibiting osteoclast-mediated bone resorption [35, 36]. Accumulating evidence shows that zoledronic acid has direct anti-tumor activity, including the capacity to inhibit cancer cell growth and survival, and the potential to synergy with anticancer therapies [37, 38]. Furthermore, translational studies have shown that zoledronic acid induces an anticancer immune response, decreases the persistence and number of disseminated tumor cells in bone marrow, and reduces the circulating levels of angiogenic growth factors [39, 40]. In addition to reducing osteolysis and preserving bone, zoledronic acid has shown anticancer activity during adjuvant therapy for breast cancer in three large clinical trials [12, 13, 41]. The AZURE [41] and ABCSG-12 trials [13] were designed as anticancer studies with disease-free survival (DFS) as the primary endpoint, whereas the effects on disease outcomes were examined as a secondary endpoint in the ZO-FAST trial [12]. The ABCSG-12 and ZO-FAST trials clearly support the potential anticancer activity of zoledronic acid. The AZURE study results did not show any benefits in terms of invasive DFS or overall survival (OS) with the addition of zoledronic acid in premenopausal and perimenopausal patients, the patient characteristics as well as the choice of treatment may influence the potential of zoledronic acid to provide clinical benefits in premenopausal patients.

Being a nitrogen-containing bisphosphonate, zoledronic acid also inhibits the activity of farnesyl diphosphate synthase, a key enzyme in the mevalonate pathway, resulting in reduced synthesis of small GTPases such as Ras, Rho and Rac [36, 42]. Previous studies have demonstrated that Ras activates HIF-1α via the Raf/MEK/ERK pathway [18]. We have ever treated an endocrine resistant patient who presented resistance after 32 months neo-adjuvant endocrine therapy. IHC analysis showed that HIF-1α expression was significantly increased in the residual specimen after letrozole treatment, while after zoledronic acid treatment due to severe osteoporosis, the expression of HIF-1α and the Ki-67 index was significantly decreased. Therefore, we postulated that zoledronic acid plays an essential role in endocrine resistance via the Ras/ERK/HIF-1α pathway.

Zhao et al. found that the HIF-1α inhibitor, PX-478 enhanced the anti-tumor effect of gemcitabine, a first-line chemotherapeutic drug for advanced pancreatic cancer, in pancreatic ductal adenocarcinoma [43]. Our study showed that inhibition of HIF-1α by zoledronic acid improved the sensitivity to endocrine therapy in breast cancer. Therefore, HIF-1α inhibitor should be considered for development as a therapeutic agent for overcoming endocrine resistance in ER- positive breast cancer.

In conclusion, the findings of this study suggest that HIF-1α is a crucial determinant of endocrine resistance in human breast cancer. Targeting of HIF-1α by zoledronic acid, a drug previously used in the prevention or treatment osteoporosis, has the potential to reverse or prevent anti-estrogen resistance *in vitro* and *in vivo*. The novel combination of zoledronic acid and endocrine therapy may offer a new therapeutic option for patients with recurrent breast cancer. Further clinical studies involving the combination of zoledronic acid and endocrine therapy in recurrent breast cancer are warranted.

## METHODS

### Patients and treatment

In our ongoing clinical trial (Supplementary Figure 1) [34], postmenopausal women with stages II–IV ER-positive (immunohistochemistry score ≥ 4) primary breast cancer were considered eligible for inclusion. After a diagnostic core needle biopsy, the patients were assigned to endocrine therapy with letrozole (Femara) 2.5 mg daily. Therapeutic breast surgery (quadrantectomy or modified radical mastectomy in association with sentinel node biopsy or axillary node dissection) was preformed after at least 4 months of primary endocrine therapy or until disease progression. This study was approved by the local Ethical Committee. Written informed consent was obtained from all patients before commencing any of the medical procedures. Tumor response was assessed by both CT and ultrasound imaging according to the WHO criteria. In a subgroup of patients, one transit dose of zoledronic acid (4 mg) was planned 4 weeks before surgery.

### Sample collection and immunohistohemical (IHC) staining

Paired breast cancer specimens from both baseline core needle biopsy and post-treatment surgery or follow-up core needle biopsy after primary endocrine therapy were collected from each patient for immunohistochemical (IHC) analysis. In the subgroup of patients who received zoledronic acid, additional tissue samples were collected after 4 weeks of adding zoledronic acid. To eliminate ischemia-induced hypoxia, samples for HIF-1α evaluation were fixed in 4% buffered formalin within 10 min of separation from their blood supply. Each set of slides was stained with commercially available antibodies. An ERα (Santa Cruz Biotechnology, TX, USA) antibody was used at 1:35 dilution, with a 10-min high-temperature antigen retrieval in citrate buffer (pH = 6.0). HIF-1α (EPITOMICS, Burlingame, CA, USA) and Ki-67 (DAKO, Carpinteria, CA, USA) antibodies were used at 1:100 and 1:150 dilution, respectively, with a 15-min high-pressure antigen retrieval in citrate buffer (pH = 6.0). Immunoreactivity was detected by using the EnVision+ System (DAKO) with diaminobenzidine chromogen according to the manufacturer's protocol. Known positive and negative controls (obtained by omission of primary antibodies) were used as a quality control of the staining. All IHC slides were examined by light microscopy by two observers blinded to patient outcome. HIF-1α levels were assessed within the entire tumor section with a semi-quantitative scale that combined proportional expression (scored as 0, no expression; 1, < 10%; 2, 10%–50%; 3, 50–80%; or 4, > 80% of cells showing nuclear staining) and staining intensity (scored as 0, none; 1, weak; 2, intermediate; or 3, strong) to obtain a total IHC score ranging from 0 to 7 [20]. Ki-67 expression was scored as the percentage of positively stained cells among 1,000 malignant cells. For ERα evaluation, the Allred score [44] was adopted and calculated.

### Cell culture and treatments

All cell lines were obtained from the American Type Culture Collection (ATCC) and maintained in RPMI 1640 supplemented with 10% fetal bovine serum plus 5% *penicillin/streptomycin* at 37°C under 5% CO_2_. The intermittent hypoxic cell line MCF-7/hyp, which was subjected to hypoxia-reoxygenation, with each cycle comprising hypoxia extending for 64 h and reoxygenation for 8 h, was cultured in an intermittently hypoxic environment as previously reported [45] for at least 3 months. In detail, cells were seeded into a 25 cm^2^ flask and exposed to hypoxic conditions in a 37°C hypoxic incubator (Thermo Electron Corporation) filled with 94.8% N2 and 5% CO_2_ to maintain oxygen levels at 0.2 to 0.5%. During the reoxygenation period, culture media were replenished under sterile conditions. After 8 h of reoxygenation, cell culture flasks were then returned to the hypoxic chamber and gradually returned to hypoxic conditions. Fulvestrant and zoledronic acid were kindly provided by AstraZeneca and Novartis (Basel Switzerland), respectively. The selective proteasome inhibitor MG-132 was purchased from Sigma. Cells were treated with drugs at the indicated final concentrations.

### Vector construction

The human full-length cDNA of HIF-1α (NM_001530.3) was obtained from Genesent. Ligation of the amplified fragments with the vectors was achieved by homologous recombination using the In-Fusion HD cloning kit (TaKaRa). The primers used to amplify the fragments were: 5′-TAGAGCTAGCGAATTATGGAGG GCGCCGGCGGCGCGAA-3′ (forward); 5′-AGATCC TTCGCGGCCTCAGTTAACTTGATCCA-3′ (reverse). Lentiviral transfer plasmids (pLKO.1) harboring shRNAs were from Sigma. The sequences of the two different shRNAs designed to target HIF-1α are as follows: 5′-CCGGCCAGTTATGATTGTGAAGTTACTCGAGTA ACTTCACAAT CATAACTGGTTTTT-3′ (sh-1), 5′-CC GGGTGATGAAAGAATTACCGAATCTCGAGATTCG TAATTCTTTCATCACTTTTT-3′ (sh-2). The control vector was a corresponding scrambled shRNA with the following sequence: 5′-CCGGCAACAAG ATGA AGAGCACCAACTCGAGTTGGTGCTCTTCATCTTGT TGTT-3′ (scramble).

### Transfection and lentivirus transduction

The lentiviral expression and control vectors were packed into HEK 293T cells to generate the corresponding lentiviruses. Transfections were performed using PEI (Polyethylenimine). MCF-7 cells infected with HIF-1α or vector control lentiviruses (designated MCF-7/HIF-1α or MCF-7/vector, respectively) and shRNAs or scramble control lentiviruses were selected and maintained in the same medium containing 2 μg/mL of puromycin (Sigma). The lentivirus-free cells were completely eradicated by puromycin selection for 72 h. The surviving lentivirus-transfected cells were identified by Western blot analysis of HIF-1α expression.

### Colony formation and cell proliferation assays

For the colony formation assay, wild-type MCF-7 cells, intermittent hypoxic cells, MCF-7/HIF-1α and MCF-7/vector cells were seeded into six-well plates and treated with fulvestrant (0.1 nM) with or without zoledronic acid (100 μM). Medium was replaced every 3 to 4 days. After 11 days, adherent cells were fixed in 10% formaldehyde for 20 min and then stained with 0.1% crystal violet for 30 min. The surviving colonies consisting of 50 or more cells were counted.

Cell proliferation assays were performed using a Cell Counting Kit-8 (CCK-8) (Dojindo, Kumamoto, Japan) according to the manufacturer's instructions. Briefly, MCF-7/vector and MCF-7/HIF-1α cells were seeded in 96-well plates (5000 cells per well) and were treated with fulvestrant and zoledronic acid for different periods of time (0, 24, 48, 72, 96 and 120 h). CCK-8 solutions were added to each well, and the plates were incubated for 3 h at 37°C. Absorbance (A) was measured at 450 nm with a microplate reader and normalized to the value of untreated cells. A duplicate plate of untreated cells was measured at 24 h.

### RNA extraction and real-time PCR

Real-time PCR was used to determine HIF-1α transcript levels in MCF-7 cells under hypoxic and normoxic conditions following treatment with or without zoledronic acid. Briefly, total RNA was isolated using TRIZOL Reagent (Life Technologies, Gaithersburg, MD) and treated with DNase I before RT-PCR analysis of HIF-1α and β-actin mRNA levels using the One-step RT-PCR Kit (QIAGEN, Valencia, CA) with HIF-1α specific primers (forward primer: 5′-TCACCACAGGACAGTACAGGATGC-3′; reverse primer: 5′-CCAGCAAAGTTAAAGCATCAGGTTCC-3′) and β-actin-specific primers (forward primer: 5′-GTACCACTGGCATCGTGATGGACT-3′; reverse primer: 5′-CCGCTCATTGCCAATGGTGAT-3′). All primers were synthesized at Sangon Biotech (Shanghai, China). Real-time PCR was performed using human-specific primers and SYBR Premix Ex Taq (Takara Bio Inc., Otsu, Japan) on a CFX96 Real-time PCR system (Bio-Rad, Hercules, CA, USA) according to the manufacturers’ instructions. For each primer pair, annealing temperature was optimized by gradient PCR. The expression (E) of each target mRNA relative to β-actin mRNA was calculated based on the cycle threshold (Ct): E = 2^−Δ(ΔCt)^, in which ΔCt = Ct _target_ – Ct_β-actin_ and Δ(ΔCt) = ΔCt_treatment_ – ΔCt_control._ Reactions without the addition of RNA samples were used as negative controls. Melt curve analyses confirmed that all real-time PCR products were produced as a single DNA duplex.

### Western blot analysis

Cells were grown in 60 mm dishes and treated with zoledonic acid for the indicated concentrations and periods of time. Cells were washed twice with ice-cold PBS and scraped into ice-cold radioimmunoprecipitation assay lysis buffer containing 50 mM Tris (pH 6.8), 20 mM EDTA, 5% sodium dodecyl sulfate (SDS), 5 mM β-glycerophosphate, and protease inhibitors (Boehringer Mannheim, Indianapolis, IN, USA). Xenograft tissues and cells were lysed in accordance with standardized protocols. Protein lysates (50 μg) were resolved by SDS-PAGE, and electrophoretically transferred to nitrocellulose membranes (Bio-Rad Laboratories, Hercules, CA, USA). After blocking in 5% BSA, membranes were hybridized overnight at 4°C with primary antibodies specific for the detection of HIF-1α (BD Transduction Laboratories), phosphorylated mitogen-activated protein kinase (MAPK) 44/42, phosphoinositide 3-kinase (PI3K/p110α), phosphorylated Akt (Ser473), phosphorylated mTOR (Cell Signaling Technology, Danvers, MA, USA), RAS (EPITOMICS) and β-actin (Proteintech). Mouse and rabbit horseradish peroxidase–conjugated secondary antibodies (Amersham Biosciences) were used at 1:5,000 dilution in TBS-Tween solution. Protein-antibody complexes were detected by chemiluminescence with the SuperSignal West Dura Extended Duration Substrate (Millipore corperation, Billerica, MA, USA), and images were captured with an ImageQuant™ LAS 4000 camera system. The experiments were repeated at least three times.

### Xenograft establishment and treatment

Mice were maintained and treated in accordance with established guidelines and the protocol was approved by an internal animal protocol review committee. Female nude BALB/c athymic nude mice (aged 6–8 weeks) were purchased from Harlan Laboratories (Italy), and were housed in air-filtered laminar flow cabinets with a 12-h light cycle and food and water ad libitum. Mice were handled using aseptic procedures and allowed to acclimatize to local conditions for one week before the experimental manipulations. A 0.72-mg-90-day–release-17β-estradiol pellet (Innovative Research, USA) was implanted subcutaneously into each mouse one week before injection. MCF-7/vector and MCF-7/HIF-1α cells (1 × 10^7^) were resuspended in PBS, mixed with Matrigel (1:1; BD Biosciences) and injected subcutaneously into the right flank of each mouse in a final volume of 200 μL. Treatment began when tumors reached an average size of 150–200 mm^3^ (i.e., in 2–4 weeks) and were thus considered as established growing xenografts. The animals were randomly allocated to receive fulvestrant (AstraZeneca, 5 mg/kg adminisered subcutaneously, twice per week) or fulvestrant followed by zoledronic acid (Novartis,120 microg/kg given subcutaneously, twice per week). Tumor xenografts were measured with calipers twice a week, and tumor volume was determined using the formula: [(Length × Width^2^)/2]. At the end of experiments, the animals were anesthetized with a 1.5% isofluorane-air mixture and sacrificed by cervical dislocation. Tumors were harvested and flash-frozen in liquid nitrogen or fixed in 10% formalin prior to paraffin-embedding. Frozen tumors were homogenized using the TissueLyser II (Qiagen). Tumor lysates were prepared, subjected to SDS-PAGE, transferred to nitrocellulose and analyzed by immunoblotting. Results are presented as mean ± standard deviation (SD).

### Statistical analysis

Values are expressed as means with 95% confidence intervals (CIs). Tumor growth curves were constructed from the mean tumor volume at each measurement time-point, with error bars representing 95% CI of the mean. The statistical significance of differences in tumor growth in the combination treatment group and in the single-agent treatment group was analyzed using the one-way analysis of variance. HIF-1α expression levels were compared between pre-treatment and post-treatment samples using the Wilcoxon test. Student's *t*-tests were used to determine the statistical significance of cellular experimental data. All analyses were performed with SPSS 17.0. *p*-value of less than 0.05 was regarded as statistically significant. All statistical tests were two-sided.

## SUPPLEMENTARY FIGURES


