# Supplementary material for: Basal and therapy-driven hypoxia-inducible factor-1α confers resistance to endocrine therapy in estrogen receptor-positive breast cancer
Source: Oncotarget. 2015 Apr 13;6(11):8648–62. doi: 10.18632/oncotarget.3257 (PMC4496173; doi:10.18632/oncotarget.3257)
Supplement: Supplementary file 1 [file oncotarget-06-8648-s001.pdf]

SUPPLEMENTARY FIGURES

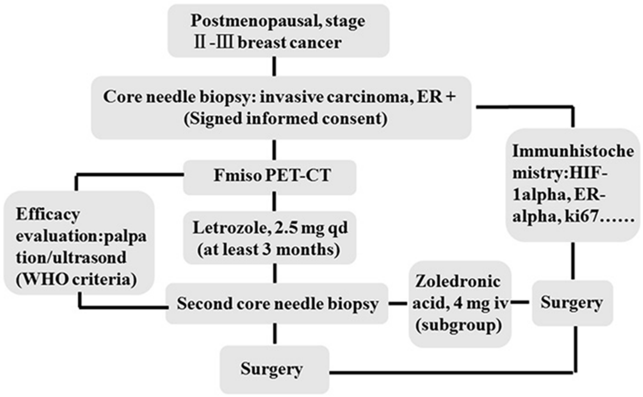

Supplementary Figure 1: Review of the ongoing clinical trial protocol. This was a single center, randomized, phase II trial. Postmenopausal women (mean age, 65.1 y; age range, 55–82 y) who had ER–positive breast cancer at stages II–IV and had notreceived prior endocrine therapy were considered eligible for this study. After a baseline biopsy, patients were assigned to primary aromatase inhibitor (AI) treatment with letrozole (2.5 mg, qd; Femara; Novartis Pharmaceuticals Corp.) for at least 3 months until a second biopsy was performed as previously described [17]. Zoledronic acid was administered once one month prior to definitive surgery. Tumor response was evaluated according to the criteria of the World Health Organization. This prospective study was approved by the local Ethics Committee. Written informed consent was obtained from each candidate patient.

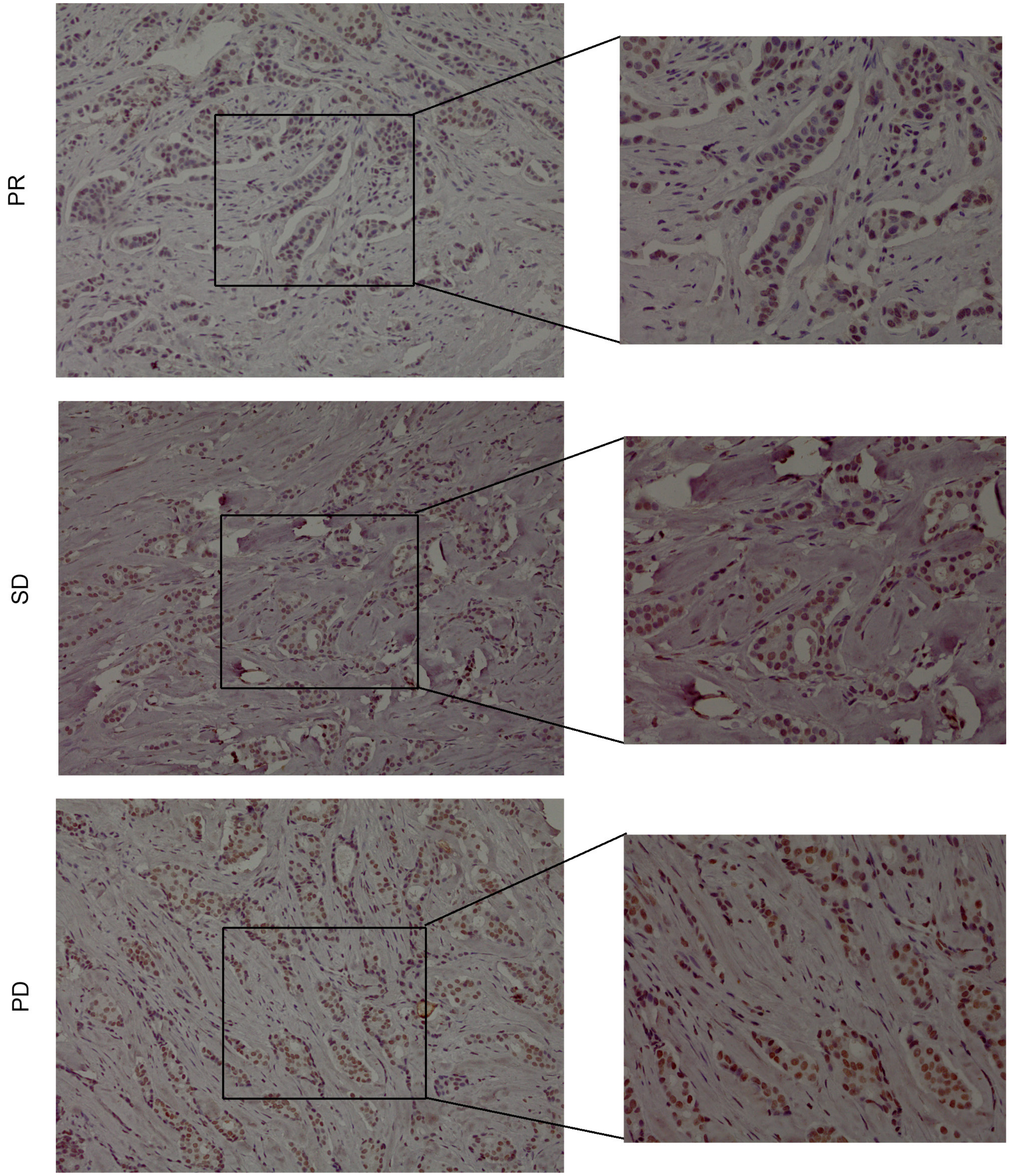

Supplementary Figure 2: Expressions of HIF-1α in tumors with no clinical response (SD, PD) and tumors with clinical response (CR, PR) by immunohistochemistry (20 × , 40 ×).
